# Primary Care Physician’s Perception and Satisfaction With Telehealth in the National Guard Primary Healthcare Centers in Jeddah, Saudi Arabia in 2022

**DOI:** 10.7759/cureus.36480

**Published:** 2023-03-21

**Authors:** Razaz Wali, Murug Shakir, Afnan Jaha, Reem Alhumaidah, Huda A Jamaluddin

**Affiliations:** 1 Family Medicine, King Abdulaziz Medical City, National Guard Hospital, Jeddah, SAU; 2 Family Medicine, King Saud Bin Abdulaziz University for Health Sciences College of Medicine, Jeddah, SAU; 3 Family Medicine, King Abdullah International Medical Research Center, Jeddah, SAU

**Keywords:** perception, satisfaction, primary healthcare centers, physicians, telemedicine, telehealth

## Abstract

Background

Telehealth is a tool to facilitate the connection between patients and their healthcare providers. With the recent emergence of telehealth, implementation of this service in primary healthcare centers (PHCs) has been accompanied by specific challenges despite the high levels of satisfaction reported. This study aimed to assess the factors that affect clinicians’ perceptions and satisfaction with telehealth in National Guard PHCs to help explore and overcome any barriers and challenges.

Methods

A cross-sectional survey was distributed among primary healthcare physicians using virtual clinics in the National Guard PHCs in Jeddah, Saudi Arabia, in 2022. A validated questionnaire from previous literature was used to evaluate clinicians’ perceptions and satisfaction with telehealth.

Results

The study included 53 primary healthcare physicians, with an overall response rate of 90%. Most physicians (77%) were satisfied with their overall experience with offering virtual visits. Nevertheless, 72% of physicians perceived patients' limited technical knowledge, and 70% considered limited access to technology a significant barrier against virtual visits. Higher satisfaction levels were significantly associated with those who did not consider the lack of integration of virtual visits with current workflow or electronic medical records (EMRs) a significant barrier to conducting virtual visits (*p*-value = 0.005).

Conclusion

Despite the undeniable advantages of telehealth, barriers, and challenges remain extant and can influence clinicians’ satisfaction. Continuous monitoring for improvements is needed to enhance the telehealth experience.

## Introduction

Telehealth is a network of applications for electronically delivering healthcare and information. It is an emergent domain concerned with overcoming barriers to communication and improving the quality of healthcare services, health outcomes, and patient satisfaction. Telehealth and telemedicine are commonly used interchangeably to refer to telecommunication in medicine. According to the Health Resources and Services Administration (HRSA), the difference between telehealth and telemedicine is their scope [1]. Telemedicine is essentially concerned with distributing remote clinical services, such as diagnosis and patient monitoring, whereas telehealth incorporates a broader range of services such as promotive, preventative, and curative care delivery. Telehealth is a block that fills the gap for providing patient care, unlimited by time and place, in exceptional circumstances such as epidemics or pandemics, lack of transportation, patient immobilization, and certain weather conditions. Telehealth can also be used as a tool to provide long-distance learning and generate virtual meetings and as a platform for physicians to depict case presentations. It also facilitates patient participation in clinical decision-making and adherence to treatment plans, improving overall health outcomes [2,3].

Telehealth was used long before the COVID-19 pandemic [4-6]. Nonetheless, a significant increase in the utilization of telehealth as a means to connect patients with their healthcare providers in a safe and physically distant manner has taken place since the pandemic [7-10]. With the recent emergence of telemedicine in the healthcare system, implementation of this service in primary healthcare centers (PHCs) has been accompanied by specific challenges despite the high levels of satisfaction reported. In a study conducted at the University of Pittsburgh Medical Center (UPMD), an 11-item, 5-point Likert survey was used to measure healthcare providers’ satisfaction with telemedicine. It concluded that 65% of healthcare providers thought the provider-patient relationship was unimpaired. In addition, more than half of the physicians enjoyed video visits and agreed they were time-saving. However, only 31% of physicians could perform a sufficient clinical examination [11]. During the COVID-19 pandemic in King Abdulaziz Medical City, Riyadh, Saudi Arabia, family medicine physicians also cited the inability to perform a physical examination as a barrier and thus preferred office-based visits [12]. Another study was conducted in community health centers (CHCs) in New York State to evaluate providers’ perceptions of telehealth and to compare different electronic modalities. Findings suggested that most clinicians agreed that telemedicine has improved patients’ access to medical care and resulted in fewer patient no-shows [13].

Additionally, most primary healthcare physicians thought telehealth had improved the monitoring of patients with chronic conditions. Nevertheless, the lack of home-based monitoring devices such as blood pressure monitors or scales presented a significant challenge in managing such cases. Also, most physicians preferred video-based visits over telephone-based appointments as they allowed family members and caregivers to engage. It also allowed for assessing the home environment and self-hygiene [3,13]. A similar finding was seen in another study in Lothian, Scotland. Most physicians agreed that visual cues from video consultations offer an advantage over telephone-based talks. However, technical difficulties, including less-than-optimal audio and image quality, were major drawbacks for many physicians [14].

Moreover, a cross-sectional study at the Seattle Veterans Affairs Primary Care Clinic evaluated physicians’ behaviors and perceived limitations toward utilizing clinical video telemedicine to home (CVTH). Findings showed that physicians were more likely to offer CVTH for patients with specific conditions such as diabetes, hypertension, and tobacco use than for others. Despite their interest in implementing CVTH in clinics, participants reported concerns, including patients’ technological incompetence and harmful interference with regular clinic flow [15]. Physicians who have conducted video visits at a large academic department of family medicine at the University of Michigan Medical School reported their experience, satisfaction, and barriers in an anonymous online 12-question survey. Half of them were new to video visits, and most participants reported high satisfaction levels and would be open to conducting video visits again. While most participants agreed that video visits were a good substitute for in-person visits, some identified video visits as less efficient for some presenting complaints due to missed important information compared to in-person visits [16].

The benefits of telehealth cannot be denied, especially in times of crisis, such as during the COVID-19 pandemic. However, controversy still surrounds the use or transition to this modality between healthcare providers. While previous literature focused on patient perspectives on virtual clinics, this study setting is the first of its kind to implement telehealth and video-based consultations in National Guard PHCs in Saudi Arabia. We plan to integrate the factors that affect clinicians’ perceptions and satisfaction with telehealth in this region to help explore and overcome any barriers and challenges that come with implementing new modalities.

## Materials and methods

This cross-sectional quantitative study was conducted in the National Guard PHCs in Jeddah, Saudi Arabia, in 2022. The included centers were Iskan Clinic, Specialized Polyclinic, Bahraa, and Shareae PHCs. The inclusion criteria of the study subjects were primary healthcare physicians using virtual clinics based on telephone, email, secure messaging, text messaging, or video appointments. This study included all 53 eligible practitioners at National Guard PHCs who utilized telehealth to provide patient care.

A 19-question survey was distributed via email to healthcare providers in the National Guard PHCs in Jeddah to fill out after obtaining their consent. The validated survey was developed by Mohammed HT et al., and the wording was modified to fit the setting to evaluate providers’ satisfaction with telehealth [17]. The survey contained 19 questions, including eight about demographics. The questions were in a multiple-choice format, and the participants were asked to select all that applied to them for some questions. The questions enquired about the frequency of use, platforms, barriers, challenges, supports, and physicians’ overall satisfaction with telehealth.

Data were entered using Microsoft Excel and coded and analyzed using Statistical Product and Service Solutions (SPSS) (IBM SPSS Statistics for Windows, Version 23.0, Armonk, NY). To test the relationship between variables, quantitative data were presented as mean and standard deviation (Mean ± SD), and categorical data were presented as frequency and percentages. Additionally, the chi-squared test was used, and a p-value of 0.05 or lower was considered statistically significant.

Approval from the Institutional Review Board (IRB) Ethical Committee at King Abdullah International Medical Research Center (KAIMRC) was obtained (ID number NRJ22J/022/01). Confidentiality of the data was maintained by ensuring it was only accessible by investigators, and the names of participants were anonymized.

## Results

Fifty-three physicians completed the questionnaire, with an overall response rate of 90%. More than half the participants were females (58%, n = 31). In addition, most respondent physicians practiced in groups (70%, n = 37), and 30% (n = 16) of participants practiced as solo physicians. Moreover, most physicians (75%, n = 40) worked in specialized polyclinics, and the rest practiced in other NGHA centers. There were 60% (n = 32) of respondents practiced in Saudi Arabia for more than five years, but only 43% (n = 23) used electronic medical records (EMRs) for more than five years. BestCare was the platform used by all participants (100%, n = 53) for virtual visits (Table 1).

**Table 1:** Demographics (N=53).

Demographics		n	%
Gender	Male	22	42%
	Female	31	58%
Practice size	Solo physician/practitioner	16	30%
	Group of physicians	37	70%
Practice geography type	Urban	50	94%
	Rural	3	6%
NGHA center practice	Specialized Polyclinic	40	75%
	Other NGHA clinics	13	25%
Practice in Saudi Arabia?	Under 5 years	21	40%
	Above 5 years	32	60%
Using Electronic Medical Records	Under 5 years	30	57%
	Above 5 years	23	43%
Products or platforms for Virtual Visits	Best Care	53	100%

**Figure 1:**
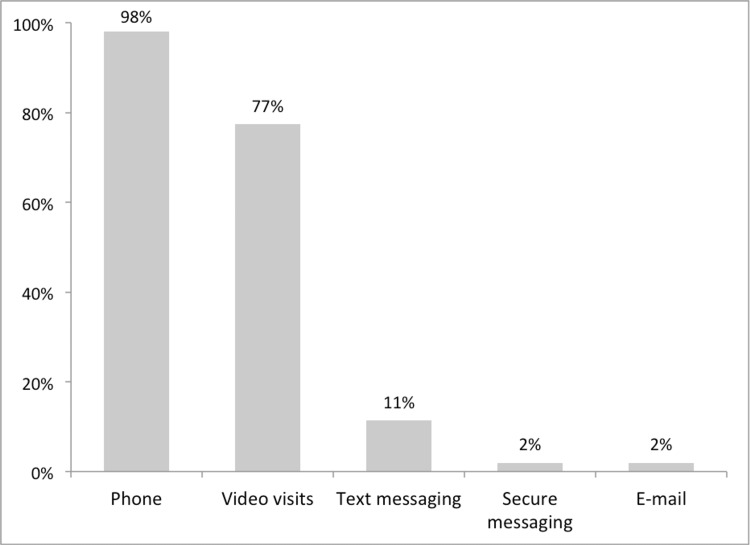
Percentages of the utilization of different kinds of technical modalities offered for virtual visits (N=53).

**Figure 2:**
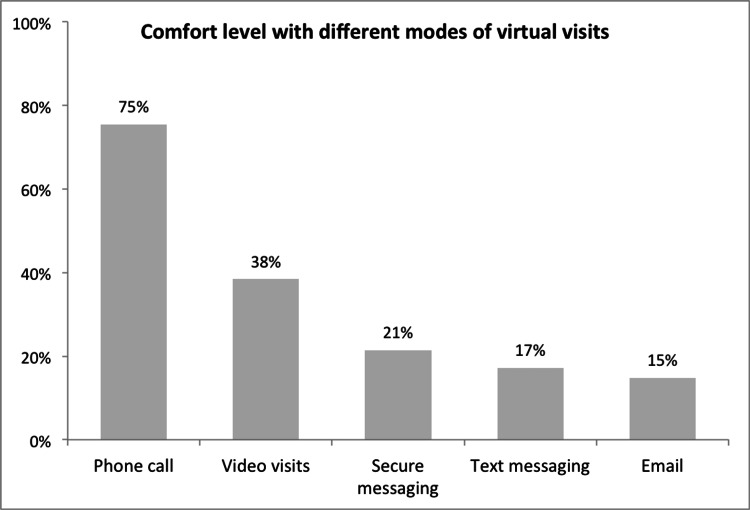
Physicians' comfort level with different modes of virtual visits (N=53).

As illustrated in Figures 1-2, almost all the respondent physicians (98%, n = 52) used phone calls to offer virtual visits, with an overall comfort level of 78% (n = 41) of participants who used video calls for their virtual clinics, and 38% (n = 20) thought that video calls were comfortable to use. In addition, text messaging (11%, n = 6), secure messaging (2%, n = 1), and emails (2%, n = 1) were used by a minority of physicians, with an overall comfort level of 17%, 21%, and 15%, respectively.

Table 2 demonstrates physicians’ overall comfort level, experience, and satisfaction. Most physicians (77%, n = 41) were satisfied with their overall experience with offering virtual visits. Nevertheless, 60% (n = 32) of participants integrated virtual visits within their day-to-day workflow poorly or moderately well, and only 40% (n = 21) of them could integrate it well.

**Table 2:** Comfort with virtual visits and technology (N=53). EMR: electronic medical records

Comfort Level with Offering Virtual Visits		n	%
Overall comfort level with using technology in general	Average	28	53%
	Expert/Advanced	25	47%
Comfort level with your current EMR	Beginner/Average	24	45%
	Expert/Advanced	29	55%
Overall experience with offering virtual visits	Satisfied	41	77%
	Neutral/Dissatisfied	12	23%
Integration of virtual visits within your day-to-day workflow	Very well	21	40%
	Moderately well to Poorly	32	60%

**Figure 3:**
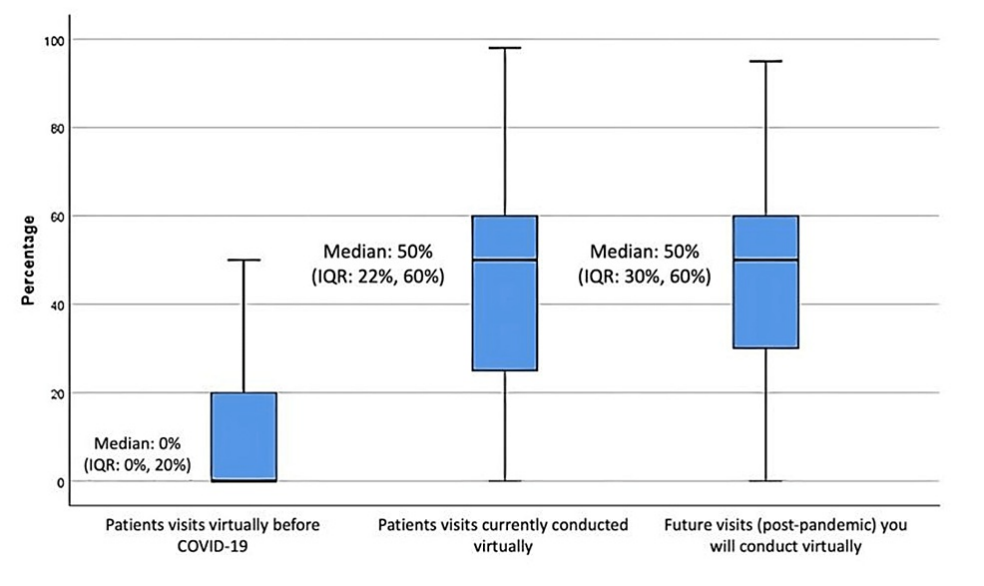
Proportion of patient visits conducted virtually before, during, and after the COVID-19 pandemic (N=53).

The proportion of patient visits conducted virtually before, during, and after the COVID-19 pandemic is shown in Figure 3. The median value was 0% (Interquartile Range (IQR): 0%, 20%) for visits conducted virtually before the pandemic. This increased to 50% (IQR: 22%, 60%) during the pandemic, according to the responses received from the participants. In addition, the expected median value for virtual visits in the future was also stated to be 50% (IQR: 30%, 60%).

When physicians were asked about any support they accessed and found useful to help them with integrating virtual visits into their practice, approximately half of them (53%, n = 28) agreed that the support they received from local colleagues who were using the technology was helpful. In addition, in-house organizational support, including IT support, was only useful for less than half the participants (42%, n = 22). Moreover, only 34% (n = 18) of respondents thought that change management supports (e.g., workflow integration, defining roles in the team) and technical training on using tools (webinars, recorded videos, one-on-one support, etc.) were helpful (Table 3).

**Table 3:** Support for integrating virtual visits in practice. PHC: primary healthcare center

Types of Support Offered to PHC Physicians for Virtual Visits	n	%
Local colleague support	28	53%
In-house organizational supports	22	42%
Change management supports	18	34%
Technical training on using tools	18	34%
Written information about how to integrate the tool into the workflow	15	28%
Evidence about the effectiveness of the tool	11	21%
Virtual care standards outlined by my profession's college	10	19%
Written resource on comparison of virtual visit platforms	8	15%
Other	2	4%

Regarding barriers and challenges, most participants (74%, n = 39) had concerns about patients overusing services. In addition, 72% (n = 38) of physicians perceived patients’ limited technical knowledge, and 70% (n = 37) considered limited access to technology or devices a significant barrier against virtual visits. Moreover, connectivity issues were a major challenge experienced by 60% (n = 32) of respondents. Additionally, 32% (n = 17) of physicians had concerns about patient privacy, and 30% (n = 16) of participants found inadequate administrative support to be a barrier to virtual visit conduction. Lack of integration with the current workflow challenged 19% (n = 10) of respondents (Table 4).

**Table 4:** Barriers/challenges experienced with virtual visits. PHC: primary healthcare center

Barriers/Challenges Faced by PHC Physicians with Conducting Virtual Visits	n	%
Concerns about patients overusing services	39	74%
Patients' limited technical knowledge	38	72%
Patients' limited access to technology/devices	37	70%
Connectivity issues	32	60%
Concerns about increase in demands on time	31	58%
Concerns about patient privacy	17	32%
Adequate administrative support	16	30%
Obtaining email consent	15	28%
Adequate training/education	11	21%
Lack of integration with current workflow or electronic medical records	10	19%
Unable to justify the cost	8	15%
Other	6	11%

Table 5 shows the satisfaction levels compared between the demographic variables, and there was no significant difference in the satisfaction levels for any of the demographic categories. Similarly, Table 6 shows there was no significant association in the satisfaction levels with offering virtual visits with the comfort level of using technology, EMRs, and integration of virtual visits with daily workflow.

**Table 5:** Association of demographic characteristics with an overall experience of offering virtual visits. ^a^ Chi-Square test ^b^ Fisher's Exact test

Demographics		Total	Overall Experience with Offering Virtual Visits				p-value
			Satisfied		Neutral/Dissatisfied		
			n	%	n	%	
Gender	Male	22	16	73%	6	27%	0.52^b^
	Female	31	25	81%	6	19%	
Practice size	Solo physician/practitioner	16	12	75%	4	25%	>0.99^b^
	Group of physicians	37	29	78%	8	22%	
Practice geography type	Urban	50	39	78%	11	22%	0.55^b^
	Rural	3	2	67%	1	33%	
NGHA center affiliation	Specialized Polyclinic	40	30	75%	10	25%	0.71^b^
	Other NGHA clinics	13	11	85%	2	15%	
Years of practice	Under 5 years	21	17	81%	4	19%	0.74^b^
	Above 5 years	32	24	75%	8	25%	
Using electronic medical records	Under 5 years	30	25	83%	5	17%	0.24^a^
	Above 5 years	23	16	70%	7	30%	

**Table 6:** Association between comfort with virtual visits and technology with an overall experience of offering virtual visits. ^a^ Chi-Square test ^b ^Fisher's Exact test EMR: electronic medical record

Comfort Level with Virtual Visits		Total	Overall Experience with Offering Virtual Visits				p-value
			Satisfied		Neutral/Dissatisfied		
			n	%	n	%	
Overall comfort level with using technology	Average	28	22	79%	6	21%	0.82^a^
	Expert/Advanced	25	19	76%	6	24%	
Comfort level with your current EMR	Beginner/Average	24	18	75%	6	25%	0.71^a^
	Expert/Advanced	29	23	79%	6	21%	
How well were you able to integrate virtual visits within your day-to-day workflow?	Very well	21	19	90%	2	10%	0.10^b^
	Moderately well to Poorly	32	22	69%	10	31%	

Table 7 also shows there was no association in the satisfaction level with the different support strategies available for the respondents. Higher satisfaction levels were significantly associated with those who did not consider the lack of integration of virtual visits with current workflow or EMRs a significant barrier to conducting virtual visits (p-value = 0.005). However, there was no significant association between the satisfaction level with any other barriers, as shown in Table 8.

**Table 7:** Association between support for integrating virtual visits in practice with an overall experience of offering virtual visits. ^a ^Chi-Square test ^b^ Fisher's Exact test PHC: primary healthcare center

Support Offered to PHC Physicians for Conducting Virtual Visits	Total		Overall Experience with Offering Virtual Visits				p-value
			Satisfied		Neutral/Dissatisfied		
			n	%	n	%	
Local colleague support	Yes	28	22	79%	6	21%	0.82^a^
	No	25	19	76%	6	24%	
In-house organizational supports	Yes	22	16	73%	6	27%	0.52^b^
	No	31	25	81%	6	19%	
Change management supports	Yes	18	14	78%	4	22%	>0.99^b^
	No	35	27	77%	8	23%	
Technical training on using tools	Yes	18	13	72%	5	28%	0.73^b^
	No	35	28	80%	7	20%	
Written information about how to integrate the tool into the workflow	Yes	15	10	67%	5	33%	0.29^b^
	No	38	31	82%	7	18%	
Evidence about the effectiveness of the tool	Yes	11	7	64%	4	36%	0.24^b^
	No	42	34	81%	8	19%	
Virtual care standards outlined by my profession's college	Yes	10	7	70%	3	30%	0.68^b^
	No	43	34	79%	9	21%	
Written resource on comparison of virtual visit platforms	Yes	8	4	50%	4	50%	0.07^b^
	No	45	37	82%	8	18%	
Other	Yes	2	1	50%	1	50%	0.41^b^
	No	51	40	78%	11	22%	

**Table 8:** Association between barriers/challenges experienced with virtual visits with an overall experience of offering virtual visits. ^a ^Chi-Square test ^b^ Fisher's Exact test PHC: primary healthcare center

Challenges Faced by PHC PHysicians When Conducting Virtual Visits	Total		Overall Experience with Offering Virtual Visits				p-value
			Satisfied		Neutral/Dissatisfied		
			n	%	n	%	
Unable to justify the cost	No	45	35	78%	10	22%	>0.99^b^
	Yes	8	6	75%	2	25%	
Concerns about patient privacy	No	36	29	81%	7	19%	0.49^b^
	Yes	17	12	71%	5	29%	
Obtaining email consent	No	38	30	79%	8	21%	0.72^b^
	Yes	15	11	73%	4	27%	
Concerns about increase in demands on time	No	22	20	91%	2	9%	0.09^b^
	Yes	31	21	68%	10	32%	
Lack of integration with current workflow or electronic medical records	No	43	37	86%	6	14%	0.005^b^
	Yes	10	4	40%	6	60%	
Adequate training/education	No	42	33	79%	9	21%	0.70^b^
	Yes	11	8	73%	3	27%	
Adequate administrative support	No	37	31	84%	6	16%	0.15^b^
	Yes	16	10	63%	6	38%	
Connectivity issues	No	21	17	81%	4	19%	0.74^b^
	Yes	32	24	75%	8	25%	
Concerns about patients overusing services	No	14	13	93%	1	7%	0.15^b^
	Yes	39	28	72%	11	28%	
Patients' limited access to technology/devices	No	16	13	81%	3	19%	0.74^b^
	Yes	37	28	76%	9	24%	
Patients' limited technical knowledge	No	15	12	80%	3	20%	>0.99^b^
	Yes	38	29	76%	9	24%	
Other	No	47	38	81%	9	19%	0.12^b^
	Yes	6	3	50%	3	50%	

## Discussion

The deployment of virtual clinics in NGHA PHCs was initiated during the COVID-19 pandemic to facilitate patient care. At that time, telehealth was the primary mode of care delivery. While previous literature focused on patient perspectives on virtual clinics, this study aimed to investigate physicians’ perceptions and satisfaction with telehealth at National Guard PHCs. The data collected in this study can also provide a better understanding of the factors influencing physicians’ satisfaction with telehealth. One of the factors affecting respondents’ satisfaction was the ability to integrate virtual visits into clinic workflow. When virtual visits are well integrated into the workflow, it decreases interference with clinic-related tasks, such as documentation, making telemedicine more time efficient. As a result, physicians will perceive telemedicine as advancing care delivery, making them feel more fulfilled.

Most participants in this study practiced in groups; however, their overall satisfaction was comparable to the physicians who practiced unaccompanied. Most of the respondents in this study utilized phone calls to conduct virtual visits, whereas secure messaging and emails were the least used forms of communication. These findings build on the existing evidence Chang JE et al. found that telephone-only visits were more commonly utilized than video-based visits since the beginning of the COVID-19 pandemic and are still the primary mode of virtual care delivery, suggesting that telephone visits strongly enable access to healthcare in the long term [13]. As opposed to the findings mentioned above, Gilson SF et al. and Fisk M et al. reported that most virtual visits were video-based rather than telephone-based [10,18]. Age, higher income, and broader access to technology are potential patient factors that can likely influence the choice between telephone or video-based visits [10,19].

Despite the advantages of telemedicine, challenges have still been noticed. Telemedicine has improved access to medical care. However, technical difficulties such as connectivity issues, lack of patient access to electronic devices, and limited technical knowledge have been reported by Katherine J et al. and Alkureishi MA et al. as barriers to video-based telemedicine, and Chang JE et al. emphasized exacerbation of such obstacles in low-income communities [3,13,16]. Glock H et al. and Barkai G et al. reported that physicians were concerned about the increased workload brought about by technical problems [20,21]. Furthermore, Samples LS et al. mentioned that providers had more concerns about their patient’s technological competency than their own [15].

Similarly, in this study, more than half of the respondents believed that patients’ inadequate technological literacy and limited access to technology or devices significantly deterred virtual visits. At the same time, all the participants conveyed average to advanced comfort levels with using technology, which is consistent with other telehealth studies [22]. Chang JE et al. also mentioned that integrating telemedicine visits with the current clinical workflow presented practical difficulties, which was also stated by one-fifth of this study’s participants [13]. On the contrary, participants in another study stated that telemedicine was integrated seamlessly within the daily clinic flow [22]. A previous study reported that an unexpected advantage to telephone visits enhanced patient privacy, as opposed to the concerns some of the physicians in this study found about reduced patient privacy [13].

Strengths, limitations, and recommendations

This was the first study of its kind conducted in National Guard PHCs in Saudi Arabia to explore the factors that affected clinicians’ perceptions and satisfaction with telehealth. This study looked into the relationship between a physician’s satisfaction and the factors that facilitate or impede the telehealth experience. In addition, the high response rate and the use of a validated questionnaire were the main strengths of our study. This study’s results may not be generalizable for all primary healthcare physicians in Saudi Arabia because it only included NGHA PHC physicians in the western region of the country. The results discovered about physicians’ perceptions and satisfaction could be considered by healthcare management and decision-makers to facilitate and improve the process of virtual clinics. More research is needed to assess patient and provider preferences regarding the modality in which to conduct virtual visits and to confirm no correlation between provider specialties and satisfaction levels.

## Conclusions

Eighty-six percent of the providers who did not think the lack of integration of virtual visits was a challenge were satisfied with the virtual visits. Other barriers, such as concerns about patient privacy, inability to justify the costs and connectivity issues, did not impact physician satisfaction. Overall physician satisfaction was not significantly associated with demographics or technology comfort levels. In addition, different support strategies available for physicians did not influence their overall experience with virtual visits. Telehealth holds the potential for improving healthcare delivery for patients and physicians, which can be achieved if constant improvements and exploration are undertaken.

## Appendices

Questionnaire

Demographic Information

1. What is your gender?*

Mark only one oval.

o  Male

o  Female

o  Prefer not to say

o  Other:

2. What is your practice size?*

Mark only one oval.

o  Solo physician/practitioner

o  Small group (2-3 physicians)

o  Medium group (4-10 physicians)

o  Large group (11+ physicians)

3. Please indicate your practice geography type.*

Mark only one oval.

o  Urban

o  Rural

4. Please indicate the NGHA center that your practice would be affiliated with.*

Mark only one oval.

o  Specialized Poly Clinic

o  Iskan Clinic

o  Bahra Clinic

o  Sharaea clinic

5. How many years have you been in practice in Saudi Arabia?*

Mark only one oval.

o  Under 1 year

o  1-5 years

o  5-10 years

o  10+ years

6. How long have you been using Electronic Medical Records (EMRs) in general?*

Mark only one oval.

o  Under 1 year

o  1-5 years

o  5-10 years

o  10+ years

7. Please indicate your comfort level with your current EMR.*

Mark only one oval.

o  Novice/Beginner

o  Average

o  Expert/Advanced

8. What is your overall comfort level with using technology in general?*

Mark only one oval.

o  Novice/Beginner

o  Average

o  Expert/Advanced

Virtual Visits

9. Do you currently offer any virtual visits (e.g., telephone, email, secure messaging, text messaging, or video appointments)?*

Mark only one oval.

o  Yes

o  No

10. What kind of virtual visits do you offer? Check all that apply.*

Tick all that apply.

o  Phone call

o  Video visits

o  Secure messaging (i.e. encrypted, PHIPA-compliant messaging platform)

o  Regular e-mail (unsecured/non-encrypted)

o  Text messaging

o  Other:

11. What is your comfort level with using different modes of virtual visits?*

Mark only one oval per row.

**Table 9:** What is your comfort level with using different modes of virtual visits?

	Very Uncomfortable	Uncomfortable	Neutral	Comfortable	Very Comfortable	Not Applicable/Not Using
Phone call						
Video visits						
Secure messaging						
Email (unsecured)						
Text messaging						

12. If you are currently offering virtual visits, what products or platforms do you use? Check all that apply.*

Tick all that apply.

o  BestCare

o  Other:

13. Thinking about all the appointments in the past week, what proportion of your patient visits do you currently conduct virtually (phone, video, secure messaging, email, text messaging)? Sliding scale: 0%-100%*

14. In general, what proportion of your patient visits did you conduct virtually before COVID-19? Sliding scale: 0%-100%*

15. In general, what proportion of future visits (post-pandemic) do you anticipate you will conduct virtually? Sliding scale: 0%-100%*

16. How well were you able to integrate virtual visits within your day-to-day workflow?*

Mark only one oval.

o  Very well

o  Moderately well

o  Poorly

17. What are the barriers/challenges that you experience with virtual visits? Check all that apply.*

Tick all that apply.

o  Unable to justify the cost

o  Concerns about patient privacy

o  Obtaining consent

o  Concerns about increase in demands on time

o  Lack of integration with current workflow or Electronic Medical Records (EMR)

o  Adequate training/education

o  Adequate administrative support (e.g., comfort with technology, insufficient administrative staff to delegate tasks, etc.)

o  Connectivity issues (e.g. inconsistent Wi-Fi/Internet connection)

o  Concerns about patients overusing services

o  Patients' limited access to technology/devices (e.g. computer/laptop/tablet, phone app)

o  Patients' limited technical knowledge

o  Other:

18. Are there any supports you accessed and found useful to help you with integrating virtual visits in your practice? Check all that apply.*

Tick all that apply.

o  Local colleague support (Connection with a local colleague who is using the technology)

o  In-house organizational supports (e.g., IT support, Data/EMR Administrators, Quality Improvement Specialists, etc.)

o  Change management supports (e.g., workflow integration, defining roles in the team)

o  Technical training on using tools (webinars, recorded videos, one-on-one support, etc.)

o  Written information about how to integrate the tool into the workflow

o  Evidence about the effectiveness of the tool

o  Virtual care standards outlined by my profession's college

o  Written resource on comparison of virtual visit platforms (cost, features, pros/cons, etc.)

o  Other:

19. What is your overall experience with offering virtual visits (e.g., telephone, email, secure messaging, text messaging, or video appointments)?*

Mark only one oval.

o  Very satisfied

o  Satisfied

o  Neither satisfied nor dissatisfied

o  Dissatisfied

## References

[1] Pruitt S (2013). The office for the advancement of telehealth. Telemed J E Health.

[2] van den Heuvel JF, Groenhof TK, Veerbeek JH, van Solinge WW, Lely AT, Franx A, Bekker MN (2018). eHealth as the next-generation perinatal care: an overview of the literature. J Med Internet Res.

[3] Alkureishi MA, Choo ZY, Lenti G (2021). Clinician perspectives on telemedicine: observational cross-sectional study. JMIR Hum Factors.

[4] Thirunavukkarasu A, Alotaibi NH, Al-Hazmi AH (2021). Patients' perceptions and satisfaction with the outpatient telemedicine clinics during COVID-19 era in Saudi Arabia: a cross-sectional study. Healthcare (Basel).

[5] (2023). Digital health and care strategy: enabling, connecting and empowering. https://www.gov.scot/publications/scotlands-digital-health-care-strategy-enabling-connecting-empowering/.

[6] (2017). Australia’s national digital health strategy. https://apo.org.au/node/182181.

[7] Calton B, Abedini N, Fratkin M (2020). Telemedicine in the time of coronavirus. J Pain Symptom Manage.

[8] Greenhalgh T, Wherton J, Shaw S, Morrison C (2020). Video consultations for covid-19. BMJ.

[9] Mann DM, Chen J, Chunara R, Testa PA, Nov O (2020). COVID-19 transforms health care through telemedicine: evidence from the field. J Am Med Inform Assoc.

[10] Gilson SF, Umscheid CA, Laiteerapong N, Ossey G, Nunes KJ, Shah SD (2020). Growth of ambulatory virtual visits and differential use by patient sociodemographics at one urban academic medical center during the COVID-19 pandemic: retrospective analysis. JMIR Med Inform.

[11] Saiyed S, Nguyen A, Singh R (2021). Physician perspective and key satisfaction indicators with rapid telehealth adoption during the Coronavirus disease 2019 pandemic. Telemed J E Health.

[12] Altulaihi BA, Alharbi KG, Alhassan AM, Altamimi AM, Al Akeel MA (2021). Physician's perception toward using telemedicine during COVID-19 pandemic in King Abdulaziz Medical City, Riyadh, Saudi Arabia. Cureus.

[13] Chang JE, Lindenfeld Z, Albert SL (2021). Telephone vs. video visits during COVID-19: safety-net provider perspectives. J Am Board Fam Med.

[14] Donaghy E, Atherton H, Hammersley V (2019). Acceptability, benefits, and challenges of video consulting: a qualitative study in primary care. Br J Gen Pract.

[15] Samples LS, Martinez J, Beru YN, Rochester MR, Geyer JR (2021). Provider perceptions of telemedicine video visits to home in a veteran population. Telemed J E Health.

[16] Gold KJ, Laurie AR, Kinney DR, Harmes KM, Serlin DC (2021). Video visits: family physician experiences with uptake during the COVID-19 pandemic. Fam Med.

[17] Mohammed HT, Hyseni L, Bui V, Gerritsen B, Fuller K, Sung J, Alarakhia M (2021). Exploring the use and challenges of implementing virtual visits during COVID-19 in primary care and lessons for sustained use. PLoS One.

[18] Fisk M, Livingstone A, Pit SW (2020). Telehealth in the context of COVID-19: changing perspectives in Australia, the United Kingdom, and the United States. J Med Internet Res.

[19] Rodriguez JA, Betancourt JR, Sequist TD, Ganguli I (2021). Differences in the use of telephone and video telemedicine visits during the COVID-19 pandemic. Am J Manag Care.

[20] Glock H, Milos Nymberg V, Borgström Bolmsjö B, Holm J, Calling S, Wolff M, Pikkemaat M (2021). Attitudes, barriers, and concerns regarding telemedicine among Swedish primary care physicians: a qualitative study. Int J Gen Med.

[21] Barkai G, Gadot M, Amir H, Menashe M, Shvimer-Rothschild L, Zimlichman E (2021). Patient and clinician experience with a rapidly implemented large-scale video consultation program during COVID-19. Int J Qual Health Care.

[22] Becevic M, Boren S, Mutrux R, Shah Z, Banerjee S (2015). User satisfaction with telehealth: study of patients, providers, and coordinators. Health Care Manag (Frederick).

